# Proteome and Transcriptome Analysis of Gonads Reveals Intersex in *Gigantidas haimaensis*

**DOI:** 10.1186/s12864-022-08407-w

**Published:** 2022-03-03

**Authors:** Yu Shi, Gaoyou Yao, Hua Zhang, Huixia Jia, Panpan Xiong, Maoxian He

**Affiliations:** 1 CAS Key Laboratory of Tropical Marine Bio-resources and Ecology, Guangdong Provincial Key Laboratory of Applied Marine Biology, South China Sea Institute of Oceanology, Chinese Academy of Sciences, 164 West Xingang Road, Guangzhou, 510301 China; 2 Southern Marine Science and Engineering Guangdong Laboratory, Guangzhou, 511458 China; 3 University of Chinese Academy of Sciences, Beijing, 100049 China

**Keywords:** Transcriptome, Proteome, Sex change, Intersex, bivalve, *Gigantidas haimaensis*

## Abstract

Sex has proven to be one of the most intriguing areas of research across evolution, development, and ecology. Intersex or sex change occurs frequently in molluscs. The deep-sea mussel *Gigantidas haimaensis* often dominates within *Haima* cold seep ecosystems, but details of their reproduction remain unknown. Herein, we conducted a combined proteomic and transcriptomic analysis of *G. haimaensis* gonads to provide a systematic understanding of sexual development in deep-sea bivalves. A total of 2,452 out of 42,238 genes (5.81%) and 288 out of 7,089 proteins (4.06%) were significantly differentially expressed between ovaries and testes with a false discovery rate (FDR) <0.05. Candidate genes involved in sexual development were identified; among 12 differentially expressed genes between sexes, four ovary-biased genes (β-catenin, fem-1, forkhead box L2 and membrane progestin receptor α) were expressed significantly higher in males than females. Combining histological characteristics, we speculate that the males maybe intersex undergoing sex change, and implied that these genes may be involved in the process of male testis converting into female gonads in *G. haimaensis*. The results suggest that this adaptation may be based on local environmental factors, sedentary lifestyles, and patchy distribution, and sex change may facilitate adaptation to a changing environment and expansion of the population. The findings provide a valuable genetic resource to better understand the mechanisms of sex change and survival strategies in deep-sea bivalves.

**Supplementary Information:**

The online version contains supplementary material available at 10.1186/s12864-022-08407-w.

## Introduction

Sex has proven to be one of the most intriguing areas of research across evolution, development and ecology [1]. Hermaphroditism, in both sequential and simultaneous forms, occurs in only ~5% of animals but is phylogenetically widespread (70% of phyla) [1, 2]. Sequential hermaphroditism can be expressed as male to female sexual transition (protandry), which is common in molluscs [3, 4].

The mechanism of sex reversal/differentiation involves both genetic and environmental factors [5, 6]. Environmental sex determination (ESD) includes sexual systems that are determined by external factors such as nutritional state, temperature, social structure, or some combination of environmental triggers. The oyster *Ostrea edulis* and other bivalves also change and reverse sex in response to nutritional and temperature cues [7]. Good nutritional conditions are favourable for the conversion of shellfish to females, while in poor nutritional conditions female *Pinctada margaritifera* undergo sex reversal to males [8]. In molluscs, sex change occurs based on local environmental factors such as population density and local mating population size and composition, as well as the age, size and nutritional status of individuals. It is thought that hermaphroditism occurs to facilitate adaptation to certain selective conditions. Sedentary lifestyles, combined with patchy distribution and environmental heterogeneity, appear to promote sequential hermaphroditism to increase reproductive output in molluscs [9].

Although the mechanisms of sex reversal are well studied in vertebrates, in invertebrates, particularly hermaphroditic marine molluscs, data on sex reversal are scarce. Based on high-throughput transcriptome, proteome and draft genome sequencing data, sex determination/differentiation is believed to be controlled by a major gene in the pacific oysters *Crassostrea gigas* [10, 11], *Chlamys farreri* [12–14], *Chlamys nobilis* [15], *Pinctada fucata* [16], *P. margaritifera* [17] and *Patinopecten yessoensis* [18], including double-sex- and mab-3-related transcription factor (DMRT) and SoxE, SOXH (SRY-like) for male sex-determining pathways, and β-catenin and fork head box L2 (foxl2) for female sex-determining pathways.

Much of our current understanding of sexual development comes from a small number of model systems, limiting our ability to make broader conclusions about the evolution of sexual diversity. Deep-sea hydrothermal vents and seeps, characterised by darkness and high concentrations of heavy metals and other toxic substances, can provide sulphide, methane and hydrogen sulphide as chemical energy for use by chemoautotrophic bacteria to support dense populations of invertebrates [19, 20]. Among the deep-sea macro-fauna, *Bathymodiolus* (Bivalvia, *Mytilidae*) mussels often dominate at many cold seep and hydrothermal vent ecosystems worldwide [19, 21]. Previous studies have focused on symbiosis [22], immunity [23], adaptation to abiotic stress [24], ecotoxicology [25], biogeography [26] and genomes [27]. The deep-sea mussel *Gigantidas haimaensis* often dominates at *Haima* cold seep ecosystems on the northwestern slope of the South China Sea [28], but knowledge on reproduction in this species is lacking.

The survival strategies through which *G. haimaensis* adapts to its environment remain poorly understood. To gain insight into the adaptive features of the gonads, we focused genes related to sex. We performed in-depth proteomics and transcriptomics analyses on gonads and analysed the impact of the environment on gonadal development in males and females. The findings expand our understanding of gonadal development in bivalves, and the influence of extreme environments on gonad development.

## Materials and Methods

### Animals and Collection

*G. haimaensis* mussels were obtained from the Haima Cold Seep (16.73°N, 110.475°E, depth 1,446 m) using the manned submersible remotely operated vehicle (ROV) *Haima* during cruise HYDZ6-202005 of the research vessel (R/V) *Haiyang 6* of the Guangzhou Marine Geological Survey (China; September 1st-6th, 2020). Upon arrival at the sea surface, some of the mussels were frozen immediately in liquid nitrogen for 24 h then stored at -80°C, while others were fixed in 100% ethanol. After cruises, mussels were placed on dry ice and transported to the South China Sea Institute of Oceanology, Chinese Academy of Sciences. Gonads were dissected for subsequent RNA extraction and histological procedures. All animal experiments were conducted in accordance with the guidelines and approval of the Animal Research and Ethics Committees of the Chinese Academy of Sciences.

### Protein Digestion

Digestion of protein (250 μg per sample) from three oysters was performed according to the FASP procedure [29–31]. Protein quality was tested by a Bradford protein assay kit according to the manufacturer’s instructions. TMT labelling of peptides was performed according to a procedure described previously [32]. Mobile phase A (2% acetonitrile, adjusted to pH 10.0 using ammonium hydroxide) and B (98% acetonitrile) were used to develop a gradient elution. The lyophilised powder was dissolved in solution A and centrifuged at 12,000 g for 10 min at room temperature. The sample was fractionated using a Waters BEH C18 column (4.6 × 250 mm, 5 μm; Waters) on a Rigol L3000 HPLC system, with a column temperature of 45°C. Eluates were monitored at an absorbance wavelength of 214 nm, fractions were collected at one tube per min, and combined into 10 fractions. All fractions were dried under vacuum, then reconstituted in 0.1% (v/v) formic acid (FA) in water.

### Liquid Chromatography Tandem Mass Spectrometry (LC-MS/MS)

Mobile phase A (100% water, 0.1% FA) and B solution (80% acetonitrile, 0.1% FA) were prepared. Samples (1 μg) were injected into a home-made C18 Nano-Trap column (4.5 cm × 75 μm, 3 μm). A home-made analytical column (25 cm × 150 μm, 1.9 μm) was employed and the column oven was set as 55°C. The separated peptides were analysed by an Orbitrap Exploris 480 instrument coupled with FAIMS (Thermo Fisher) and a Nanospray Flex electrospray ionisation (ESI) device with a spray voltage of 2.1 kV and an ion transport capillary temperature of 320°C. The data-dependent acquisition mode was employed for MS data collection, the FAIMS compensation voltage was set at -45 and -65, and the acquisition parameters were as follows: full scan ranges from *m/z* 350 to 1500 with a resolution of 60,000 (at *m/z* 200), automatic gain control (AGC) target value = Auto (the optimal capacity was automatically calculated by the software according to other parameters), and maximum ion injection time = Auto. The scan-round time for MS/MS was set to 1s, and the precursors in the full scan were selected from high to low abundance and fragmented by higher energy collisional dissociation (HCD) with a resolution of 30,000 (at *m/z* 200), the turbo TMT+precursor Fit function was turned on, and the AGC target value was 1×10^5^. The maximum ion injection time was set to Auto, the normalised collision energy was 36%, the intensity threshold was 5.0×10^3^, and the dynamic exclusion parameter was 45 s. Raw MS data were named “raw”.

### Data Analysis

Label-free quantification was carried out in MaxQuant as previously described [33]. The resulting spectra from each run were searched separately against the 720541-X101SC21041130-Z02-unigene.blast.pep.fasta database using the Proteome Discoverer 2.4 (PD 2.4) search engine (Thermo Fisher).

In order to improve the quality of analysis results, PD 2.4 software was used to further filter the retrieval results, and a credibility score >99% identified peptide spectrum matches (PSMs). The identified proteins included at least one unique peptide and all identified PSMs and proteins had a false discovery rate (FDR) <1.0%. The protein quantitation results were statistically analyzed by t-test, and quantitative differences between experimental and control groups with *p* <0.05 and fold change (FC) ≥2.0 and FC ≤0.50 were defined as differentially expressed proteins (DEPs).

The MS proteomics data have been deposited at the Science Data Bank under dataset identifier CSTR 31253.11.sciencedb.01147/ DOI 10.11922/sciencedb.01147.

### Construction of Complementary DNA Library and Illumina Sequencing

Total RNA from three oysters was used as input material for RNA sample preparation. Briefly, mRNA was purified from total RNA using poly-T oligo-attached magnetic beads. Fragmentation was carried out using divalent cations under elevated temperature in First Strand Synthesis Reaction Buffer (5×). First-strand cDNA was synthesised using random hexamer primer and M-MuLV reverse transcriptase, and RNaseH was added to degrade RNA. Second-strand cDNA synthesis was subsequently performed using DNA Polymerase I and dNTPs. Remaining overhangs were converted into blunt ends via exonuclease/polymerase activities. After adenylation of 3’ ends of DNA fragments, adaptors with a hairpin loop structure were ligated to prepare for hybridisation. In order to select cDNA fragments mainly 370-420 bp in length, the library fragments were purified with an AMPure XP system (Beckman Coulter, Beverly, USA) [34, 35]. PCR was performed using Phusion High-Fidelity DNA polymerase, Universal PCR primers, and Index (X) Primer. Finally, PCR products were purified using an AMPure XP system and library quality was assessed on a Qubit 2.0 Fluorimeter, a Agilent Bioanalyzer 2100 system, and by quantitative real-time PCR (qRT-PCR).

Clustering of index-coded samples was performed on a cBot Cluster Generation System using a TruSeq PE Cluster Kit v3-cBot-HS (Illumina) according to the manufacturer’s instructions. After cluster generation, library preparations were sequenced on an Illumina Novaseq platform and 150 bp paired-end reads were generated.

### Data Filtering and *De Novo* Assembly

Raw data (raw reads) in fastq format were processed via in-house perl scripts. In this step, clean data (clean reads) were obtained by removing reads containing adapter, reads containing N bases, and low-quality reads from raw data. Meanwhile, Q20, Q30 and GC content were calculated for clean data. All downstream analyses were based on clean data of high quality. Transcriptomes were separately assembled *de novo* using Trinity with min_kmer_cov set to 2 by default and all other parameters set to default (http://trinityrnaseq.sourceforge.net/) [34].

### Gene Functional Annotation

Gene functions were annotated using Nr (NCBI non-redundant protein sequences), Nt (NCBI non-redundant nucleotide sequences), Pfam (Protein family), KOG/COG (Clusters of Orthologous Groups of proteins), Swiss-Prot (a manually annotated and reviewed protein sequence database), KO (KEGG Ortholog database) [36] and GO (Gene Ontology) databases [37].

### Differential Expression Analysis

Differential expression analysis of two conditions/groups (two biological replicates per condition) was performed using the DESeq2 R package (1.20.0). DESeq2 provides statistical routines for determining differential expression in digital gene expression data using a model based on the negative binomial distribution. The resulting *p*-values were adjusted using the Benjamini and Hochberg’s approach for controlling the FDR. Genes with an adjusted *p*-value <0.05 and | log2foldchange | > 1 identified by DESeq2 were assigned as differentially expressed [34].

### Histological Procedures

After fixation in alcohol for 24 h, gonadal tissues were dehydrated and embedded in paraffin for histology. Tissues were serially sectioned at 7 μm and stained with hematoxylin and eosin. Classification of the sex stage was determined under light microscopy [14].

### RT-qPCR Validation

A total of 12 genes were selected for RT-qPCR validation. Gonadal tissues of *G. haimaensis* were collected from 5 male and 5 female oysters for qRT-PCR. Tissues were ground in a homogeniser (IKA, Staufen, Germany). Total RNA was isolated from whole tissues with TRIzol Reagent (Invitrogen, Carlsbad, CA, USA), quality was checked by 1.2% (w/v) agarose gel electrophoresis, and quantity was measured using a Quawell Q5000 ultraviolet spectrophotometer (San Jose, CA, USA). All RNAs were treated with DNase I to avoid genomic contamination. A 1 μg sample of isolated RNA was used to synthesise first-strand cDNA using a ReverTra Ace-a First Strand cDNA Synthesis Kit (Toyobo, Tokyo, Japan). Primers designed for each gene are listed in Table 1. qPCR was performed using a Roche LightCycler 480 RT-PCR system with SYBR(R) Premix Ex Taq (Toyobo) according to the manufacturer’s protocol. After amplification, fluorescent data were converted to threshold cycle (Ct) values. Concentrations of templates in samples were determined by relating Ct values to standard curves. Target gene transcript levels were normalised against reference gene transcript levels. Reference genes were 60s RP-L15 and β-actin [14].

**Table 1:** List of primers for quantitative PCR validation

Gene name	Pro/Tran ID	Primer sequence (5’ to 3’)
*beta-catenin*	Cluster-1780.1/Cluster-1780.1; orf1	F: AATGCTTGTATGGCTGTT R: CTGTATCTTCTTCGGGTG
*conodipin*	-/Cluster-5499.0	F: AAATAGAAGCGTCAAACG R: GTAGAGGGACCAGCCAAT
*Cyp450*	Cluster-19168.0;orf1/Cluster-19168.0	F: GGCAAAGTTCTGTCCAAT
		R: TAAGTCTAAGCAGCGTTC
*EGFC*	Cluster-18098.1/Cluster-18098.1;	F: CGTGGACAAAGCCATAAA R: CCATATTCTTCGCCCATA
*fem-1*	Cluster-10581.0;orf1/Cluster-10581.0	F: CCTGAACGGTTACACTCC R: AAATTGCCGAAACTACTG
*foxl2*	-/Cluster-22036.0	F: AATCCAGCAGCACCAACA R: TACGGGAAGGGAAACGAG
*MMP*	Cluster-4389.6602;orf1/Cluster-4389. 6602	F: CGTGCTCTTTGTGAACTT R: GGAGATACTTTGGCTTTT
*MPI*	Cluster-4389.613;orf1/Cluster-4389.613	F: GCCTATGCGATATTTCTT R: TTGTTTCTGTTCGGTTGA
*MPR*	-/Cluster-14850.0	F: TATTTGAGTGCTGGTTGA R: ATCGGTACTGGGTATGTT
*PLAC8*	-/Cluster-4389.12722	F: TGGAAACTCGGCTCAACA R: CTGGCAACATAGACAAAGAAAA
*SOX2*	-/Cluster-16149.0	F: ATGGCACCTCAAACTACAC R: AACTTGCGAAACTCCTCC
*vitellogenin*	Cluster-20609.0;orf1/Cluster-20609.0	F: AGCCAGAGGAAGTAAGGA R: TATACGAATGTGCCAACA
*60s RP-L15*	Cluster-4389.7866;orf1/Cluster-4389.7866	F: AGCATCTGACACGGAGCA R: GACACGAGCCAGCAAGAA
*β-actin*	Cluster-4389.8162/Cluster-4389. 8162	F: GATGAAGCGCAGAGCAAGAG R: TTGTGTCATCTTCTCTCTGTTTGCT

## Results

### Histological Characteristics of *G. haimaensis* Gonads

**Fig. 1:**
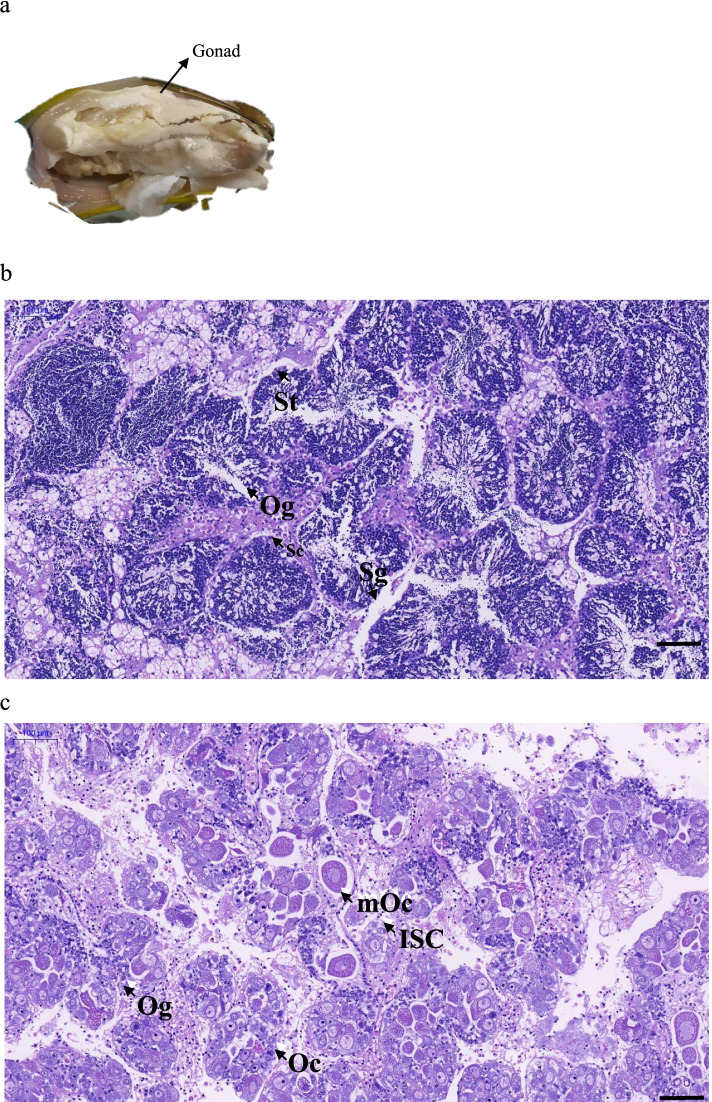
The appearance and histology of gonads in *G. haimaensis*. a. The appearance of gonads; b. Histology of male gonads; c. Histology of female gonads. ISC, intragonadal somatic cell; Og, oogonium; Oc, oocyte; mOc, mature oocyte; Sg, spermatogonium; Sc, spermatocyte; St, spermatid; Scale bar = 100 μm.

Gonads were characterised by a majority of spermatogenic cysts filled with spermatids, but some spermatogenic cysts were empty since spermatozoa may have been released. Additionally, some primary oocytes were observed between spermatogenic cysts (Fig. 1b). Ovaries were characterised by mostly mature oocytes, a few oogonia, some empty follicular cavities, and intragonadal somatic cells (Fig. 1c).

### *G. haimaensis* Gonad Transcriptome

As listed in Table 2, Illumina sequencing of ovary and testis transcriptomes generated 128,452,470 raw reads of 100 bp, of which 125,797,626 (97.93%) remained after quality filtering, and 20,966,271 filtered clean reads with Q20 >97.17% were obtained from each library. Raw reads have been submitted to the Science Data Bank under accession numbers CSTR 31253.11.sciencedb.01146/DOI 10.11922/sciencedb.01146. *De novo* Trinity assembly from combined ovary and testis reads produced 78,860 assembled transcripts and 42,238 unigenes. BUSCO revealed a transcriptome completeness of 89.4% of, indicating high-quality *de novo* assembly.

**Table 2:** Clean Data Summary

sample	raw_reads	clean_reads	clean_bases	error_rate	Q20	Q30	GC_pct
X1	21453392	20994526	6.3G	0.03	97.29	92.07	33.12
X2	20859770	20429242	6.13G	0.03	97.29	92.23	33.39
X3	21341314	20796278	6.24G	0.03	97.42	92.5	34.04
C1	21923435	21424536	6.43G	0.03	97.17	91.9	32.79
C2	20940410	20623937	6.19G	0.03	97.24	92.05	33.19
C3	21934149	21529107	6.46G	0.03	97.17	91.9	32.74

Eventually, 56.79% of 78,860 assembled transcripts were annotated using at least against one of the databases (Supplementary Table S1), and 17,090 (40.46%) had significant matches against the NR database.

**Fig. 2:**
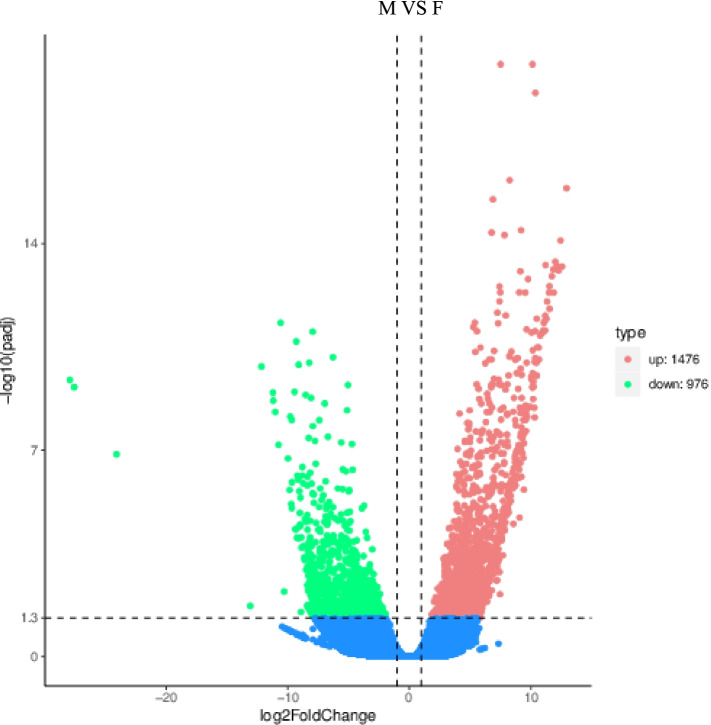
DEGs (differentially expressed genes) identified from six gonad groups. The horizontal axis represents the fold change of gene expression in different samples (log2 Fold Change), and the vertical axis represents the significance level of expression difference (-log_10_ padj). Significant differences are indicated by cutoff (-log_10_ padj > 1.3, *p* <0.05). Red indicates upregulated genes and green indicates downregulated genes among the six gonad groups.

Differential expression analysis revealed that 2,452 out of 42,238 genes (5.81%) were significantly differentially expressed between ovaries and testes with FDR <0.05. Among them, 976 (39.80%) were significantly higher in ovaries (hereafter called ovary-biased genes) compared with 1,476 (60.20%) in testes (hereafter called testis-biased genes; Fig. 2).

**Fig. 3:**
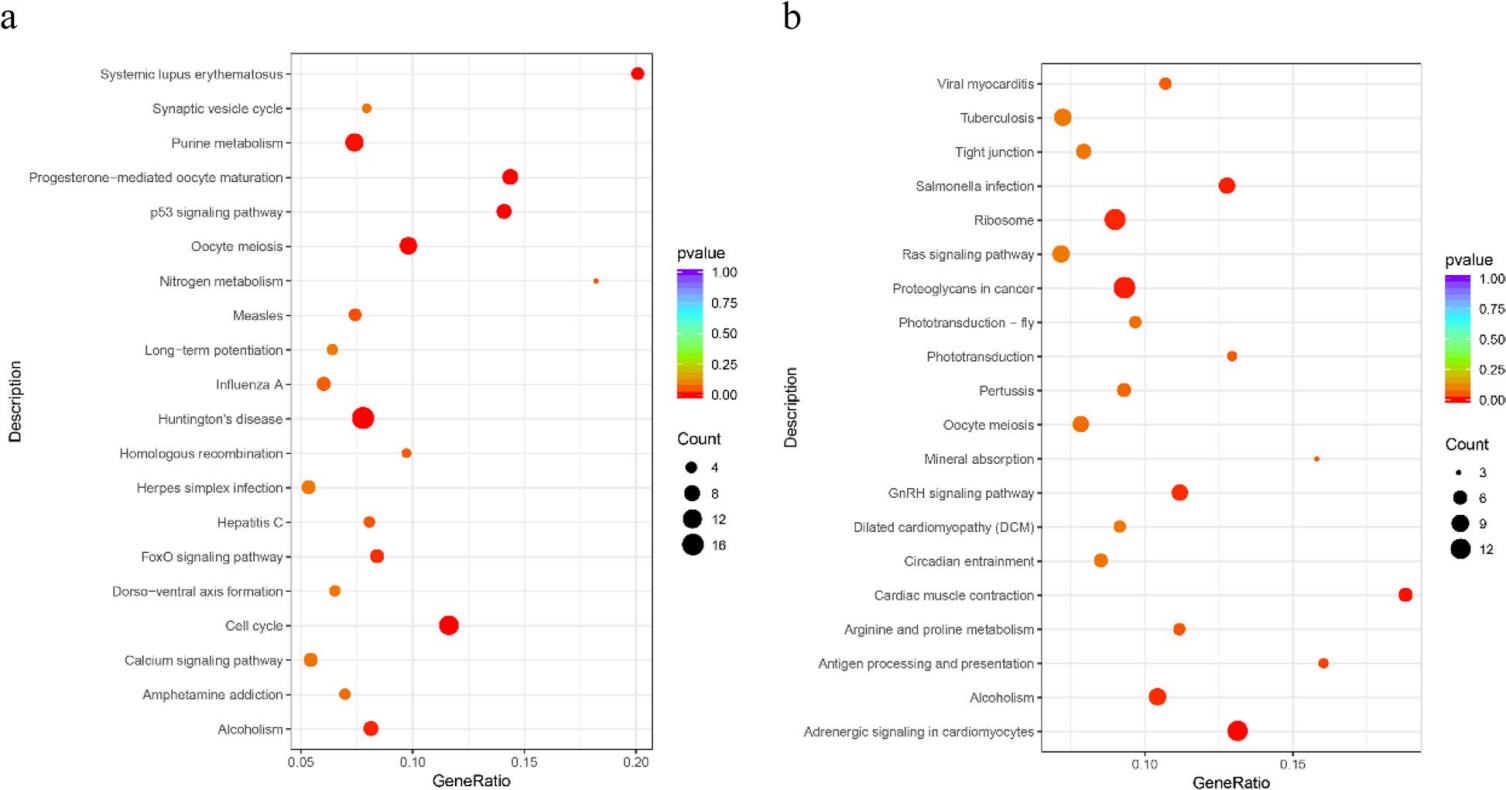
KEGG pathway enrichment scatter diagram based on the transcriptome data. The vertical axis represents the pathway name, and the horizontal axis represents the Rich factor corresponding to the pathway [38–40]. Significant differences are indicated by *p* value (*p* <0.05). The magnitude of the *p* value is represented by the colour of the dots; the smaller the *p* value, the closer the colour is to red. The number of DEGs (differentially expressed genes) within each pathway is indicated by the dot size.

All differentially expressed genes (DEGs) were subjected to GO functional analysis. Based on GO analysis, testis-biased genes were linked to 1,399 biological processes, 357 cellular components and 547 molecular functions annotated for GO term assignments, mainly related to cellular anatomical entity, membrane and organelle (Table 3). Ovary-biased genes included 1,090 biological processes, 286 cellular components and 420 molecular functions annotated for GO term assignments, mainly belonging to cellular macromolecule metabolic process, protein metabolic process and metal ion binding (Table 3). Meanwhile, testis-biased genes were associated with 161 KEGG annotation, mainly belonging to cell cycle, purine metabolism, oocyte meiosis, progesterone-mediated oocyte maturation and p53 signalling pathway (Fig. 3a, Table 4). Ovary-biased genes included 215 KEGG annotations, mainly belonging to ribosome, Ras signalling pathway, GnRH signalling pathway, oocyte meiosis and tight junction categories (Fig. 3b, Table 4). Most of these GO term and KEGG pathway enrichments are closely related to sex differentiation or determination, such as oocyte meiosis, progesterone-mediated oocyte maturation and GnRH signalling pathway.

**Table 3:** GO terms enriched in ovary- and testis-biased genes of *Gigantidas haimaensis*

ID code	Description	Term_type	DEG_item	*P* value
Ovary-biased				
GO:0044260	cellular macromolecule metabolic process	BP	120	0.049098
GO:0019538	protein metabolic process	BP	70	0.039514
GO:0046872	metal ion binding	MF	66	0.0079916
GO:0043169	cation binding	MF	66	0.0092841
GO:0055114	oxidation-reduction process	BP	36	0.045325
GO:0005509	calcium ion binding	MF	25	9.69E-05
GO:1990234	transferase complex	CC	20	0.01068
GO:0030001	metal ion transport	BP	20	0.021903
GO:0006260	DNA replication	BP	17	0.027564
GO:0065008	regulation of biological quality	BP	17	0.036641
Testis-biased				
GO:0110165	cellular anatomical entity	CC	361	0.0033264
GO:0016020	membrane	CC	200	0.044805
GO:0043226	organelle	CC	183	0.049217
GO:0043229	intracellular organelle	CC	173	0.041224
GO:0051179	localization	BP	143	0.02073
GO:0051234	establishment of localization	BP	136	0.028072
GO:0006810	transport	BP	135	0.028886
GO:0071840	cellular component organization or biogenesis	BP	97	0.025886
GO:0043232	intracellular non-membrane-bounded organelle	CC	94	0.0022039
GO:0043228	non-membrane-bounded organelle	CC	94	0.0041935

**Table 4:** KEGG pathways enriched in ovary- and testis-biased genes of *Gigantidas haimaensis*

ID code	Pathway	Genes	*P* value
Ovary-biased			
ko04261	Adrenergic signaling in cardiomyocytes	12	0.000859
ko04260	Cardiac muscle contraction	6	0.003592
ko04912	GnRH signaling pathway	8	0.014003
ko04612	Antigen processing and presentation	4	0.026682
ko00330	Arginine and proline metabolism	5	0.047621
ko04744	Phototransduction	4	0.048889
Testis-biased			
ko04110	Cell cycle	13	2.05E-05
ko04914	Progesterone-mediated oocyte maturation	8	0.000215
ko04115	p53 signaling pathway	7	0.000594
ko04114	Oocyte meiosis	10	0.000635
ko00230	Purine metabolism	11	0.003019
ko04068	FoxO signaling pathway	6	0.014706
ko00910	Nitrogen metabolism	2	0.042186

### *G. haimaensis* Gonad Proteome

To explore sex-related protein expression profiles in *G. haimaensis*, we conducted a large-scale proteomics study using label-free LC-MS/MS data. We studied gonadal tissues from six mature male (M) and mature female (F) *G. haimaensis* samples. We obtained 50,127 unique peptides and 7,089 protein groups of *G. haimaensis* proteins.

**Fig. 4:**
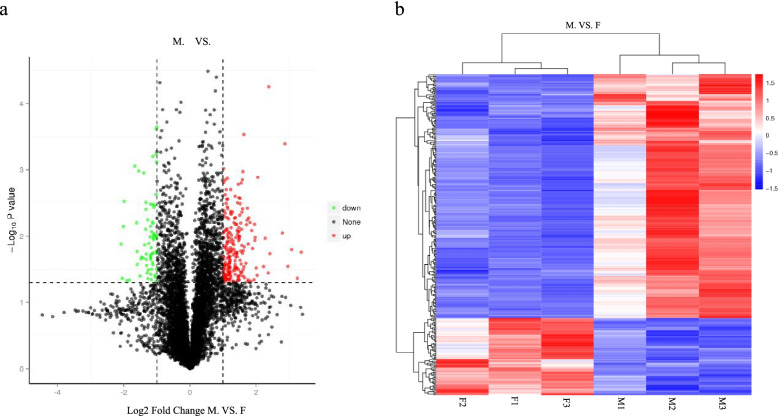
**a**. Volcano plot of DEPs. The horizontal axis represents the differential multiple (log2 value) of differential proteins, and the vertical axis represents *p* value (-log_10_ value, *p* <0.05), Black represents proteins with no significant differences, red represents upregulated proteins, and green represents downregulated proteins. **b**. Heatmap of DEP clustering. The longitudinal axis indicates the clustering of samples, and the transverse axis shows the clustering of proteins. The shorter the clustering branch, the higher the similarity.

A total of 288 DEPs were identified between M and F, with 219 (76%) upregulated in M and 69 (24%) downregulated in M (Fig. 4a, Supplementary Table S2). The expression patterns of proteins among M and F groups were quite similar (Fig. 4b).

**Fig. 5:**
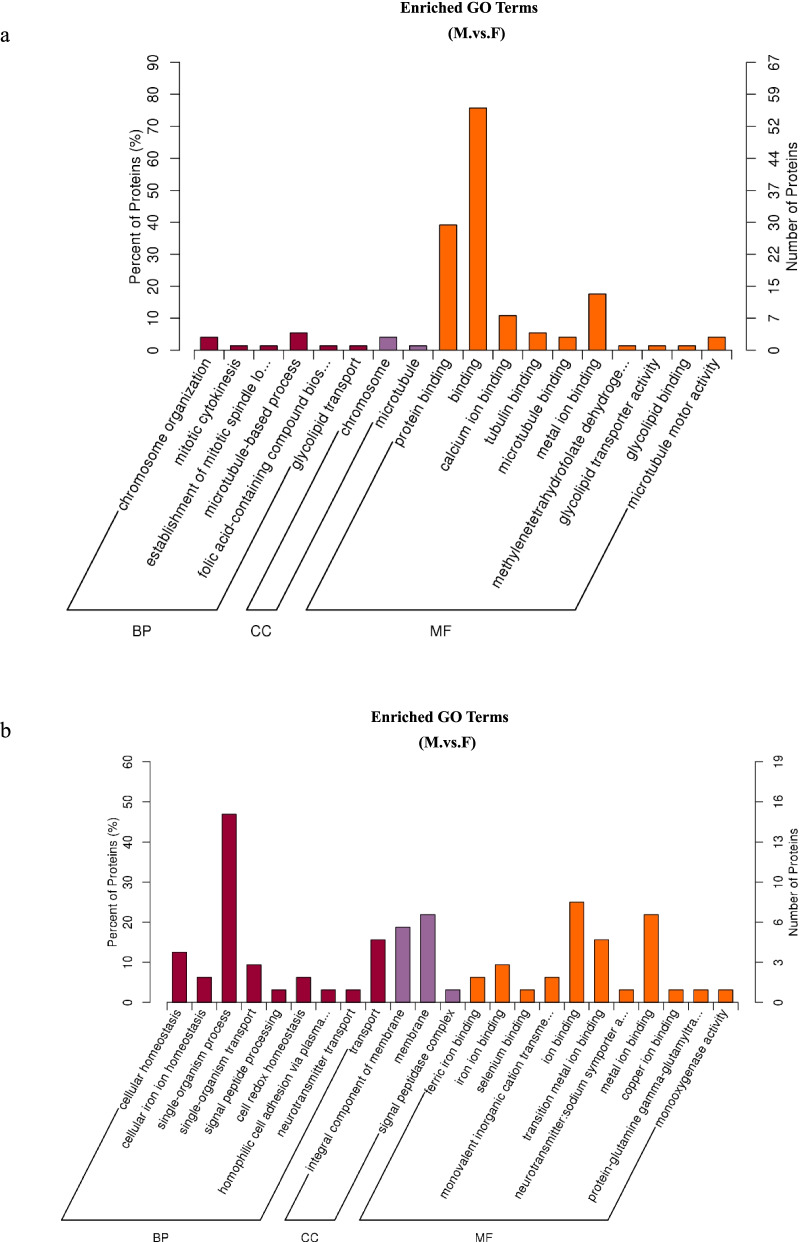
Enriched GO terms for the proteome. a. GO enrichment between DEPs of M and F groups (*p* value ≤ 0.05). b. GO terms of proteins overexpressed in F compared with M (*p* value ≤ 0.05).

We analysed GO enrichment between DEPs of M and F groups. The significantly enriched GO categories are shown in Figure 5. Numerous proteins overexpressed in M (compared with F) were enriched in 18 GO terms: 7 proteins in cellular protein modification process, 6 proteins in phosphate-containing compound metabolic process; 6 proteins in intracellular non-membrane-bounded organelle, 3 proteins in chromosome; and 56 proteins in binding, 29 proteins in protein binding (Fig. 5a, Supplementary Table S3).

Numerous proteins overexpressed in F (compared with M) were enriched in 23 GO terms: 15 proteins in single-organism process, 7 proteins in single-organism metabolic process, 7 proteins in membrane, 6 proteins in integral component of membrane, and 8 proteins in ion binding, 7 proteins in metal ion binding, (Fig. 5b, Supplementary Table S4).

**Fig. 6:**
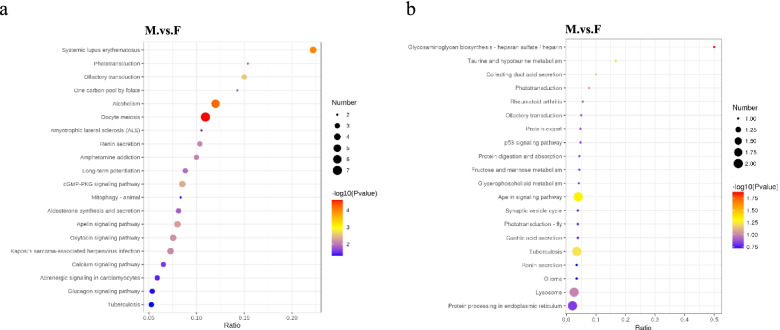
KEGG pathway enrichment scatter diagram for the proteome. The vertical axis represents the pathway name, and the horizontal axis represents the Rich factor corresponding to the pathway [38–40]. Significant differences are indicated by *p* value (*p* <0.05). The magnitude of the *p* value is represented by the colour of the dots; the smaller the *p* value, the closer the colour is to red. The number of DEGs (differentially expressed genes) within each pathway is indicated by the dot size.

Meanwhile, testis-biased genes had 84 KEGG annotations, mainly belonging to oocyte meiosis, cGMP-PKG signalling pathway, apelin signalling pathway, and oxytocin signalling pathway (Fig. 6a). Ovary-biased genes had 70 KEGG annotations, mainly belonging to metabolic pathways, apelin signalling pathway, lysosome, and protein processing in endoplasmic reticulum (Fig. 6b).

**Fig. 7:**
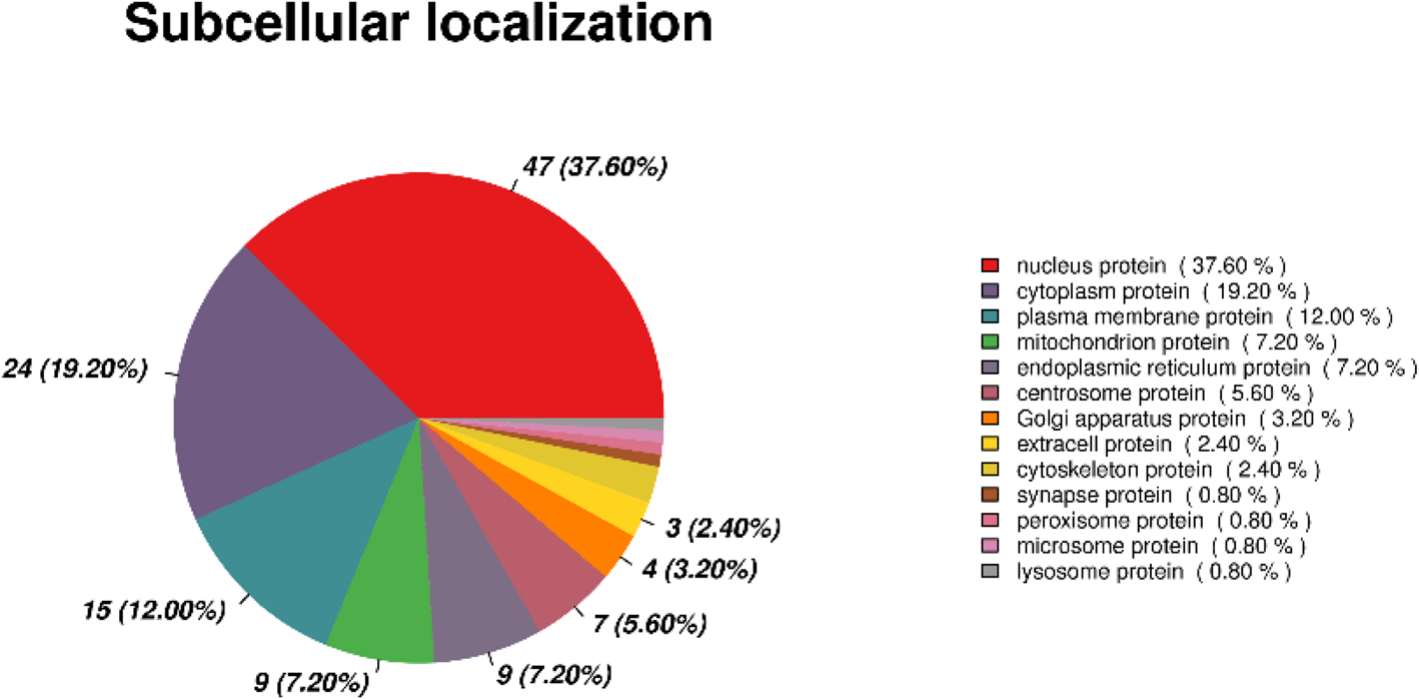
Subcellular localisation analysis of DEPs The subcellular localisation was analysed for DEPs for each comparison.

Statistical analysis of the subcellular localisation of the differentially expression proteins was performed and the results are shown in Figure 7. In total, 47 (37.60%) proteins were nuclear proteins, 24 (19.20%) were cytoplasmic proteins, 15 (12.00%) were plasma membrane proteins, 9 (7.20%) were mitochondrial and endoplasmic reticulum proteins, and 7 (5.60%) were centrosome proteins.

### Comparison of Protein and mRNA Expression Levels

**Fig. 8:**
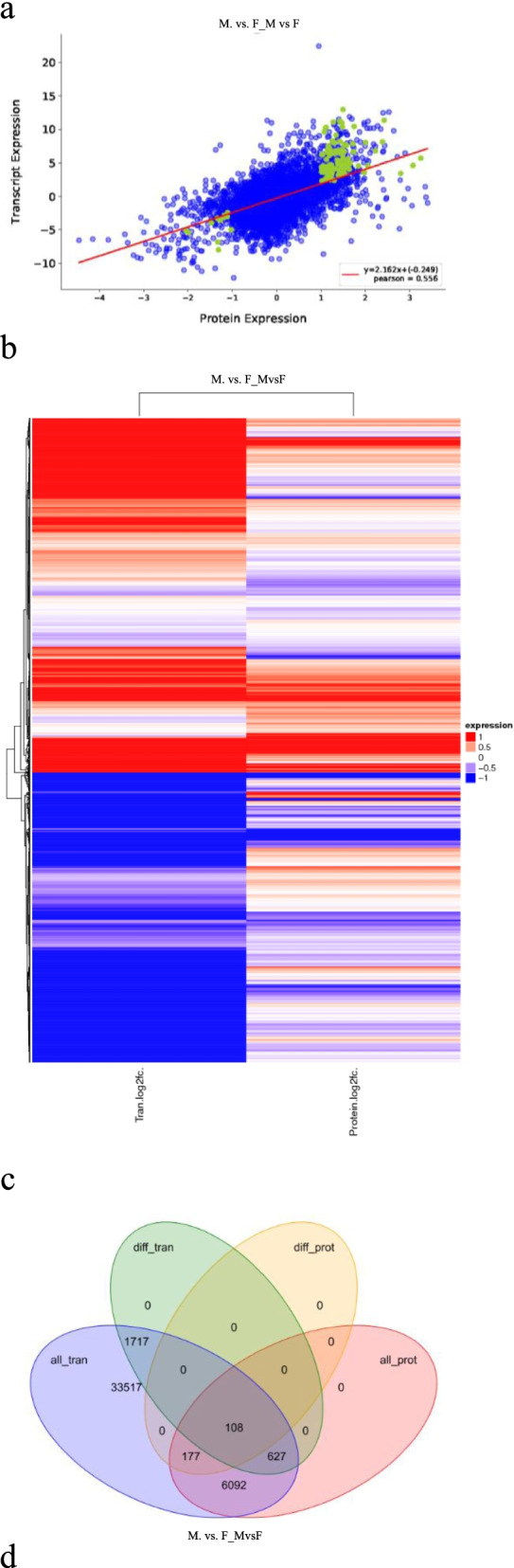
Proteome and transcriptome association analysis. a. Correlation analysis of transcriptome and proteome data. Green represents proteins with significant differences in expression (*p* < 0.05), and blue represents proteins with no significant differences in expression. The horizontal axis represents multiple differences for corresponding proteins identified from the proteome data (log2 value), and the vertical axis represents multiple differences for corresponding genes identified from the transcriptome data (log2 value). b. Heatmap of the correlation analysis of transcriptome and proteome data. Red represents upregulated genes and blue represents downregulated genes. c. Venn diagrams from the comparison of transcriptome and proteome data. all_tran, all genes identified from the transcriptome data; diff_tran, DEGs (differentially expressed genes) identified from the transcriptome data (*p* < 0.05); all_prot, all proteins identified from the proteome data; diff_prot, DEPs identified from the proteome data (*p* < 0.05). d. Correlation analysis of GO functional enrichment. Red columns represent the GO enrichment results for the proteome; grey columns represent the GO enrichment results for the transcriptome. e. Heatmap of KEGG pathway enrichment. Red represents upregulated KEGG pathways; blue represents downregulated KEGG pathways.

Pearson correlation coefficients for protein abundance measured by label-free assay and mRNA levels measured by high-throughput Illumina HiSeq 2500 sequencing gave a high correlation for F versus M (r = 0.556; Fig. 8a, b, Supplementary Table S5). We compared log2-transformed protein expression FC and mRNA expression FC values for DEPs between F and M (*p* <0.05; Fig. 8). A total of 108 differed between F and M at both protein and mRNA levels (Fig. 8c); 177 differed between F and M at the protein but not the mRNA level (Fig. 8c); 627 differed between F versus M at the mRNA but not the protein level (Fig. 8c). GO analyses of transcriptomes revealed DEGs for F versus M that were largely involved in cellular process, consistent with GO analyses of the proteome (Fig. 8d, Supplementary Table S6). The clustering heatmap for KEGG pathway enrichment based on proteome and transcriptome data showed that a large percentage of DEGs with transcriptomic KEGG pathway enrichment were also displayed proteomic KEGG pathway enrichment (Fig. 8e, Supplementary Table S6). These results indicate an overall good correlation between mRNA and protein levels among gonad genes.

### Genes Related to Sexual Development

Three selected genes (CYP450, Epidermal Growth Factor Receptor (EGFR) and fem-1) showed significant differential expression at both mRNA and protein levels, four genes (beta-catenin, MMP, MPI, vitellogenin) displayed significant differential expression only at the mRNA level, and five genes (conodipin, foxl2, membrane progestin receptor α (MPRα), PLAC8, SOX2) exhibited significant differential expression only at the mRNA level but not found in proteomic annotation (Table 5). Among DEGs identified from transcriptome data, differential expression of 12 sex-related genes was confirmed by quantitative PCR (qPCR), validating the RNA sequencing (RNA-Seq) data (Table 1).

**Table 5:** Selected sex reversal –related genes detected in transcriptome, proteome and tested by Q-PCR

Transcriptome						Proteome				
Gene	Gene_ID	Gene annotation	log2FC	p val	Comparison	Protein ID	log2FC	p val	Comparison	q-PCR
β-catenin	Cluster-1780.1	Armadillo repeat-containing protein 3 [*Mizuhopecten yessoensis*]	3.2356	9.51E-05	F < M	Cluster-1780.1;orf1	0.937422094	0.071511626	-	F < M
conodipin	Cluster-5499.0	Conodipine-M alpha chain [*Mizuhopecten yessoensis*]	6.7606	1.95E-05	F < M	-			-	F < M
CYP450	Cluster-19494.0	cytochrome P450 family 4, partial [*Mytilus galloprovincialis*]	-5.1622	0.002184	F > M	Cluster-19168.0;orf1	-1.02459691	0.000583065	F > M	F > M
EGFC	Cluster-18602.0	Epidermal growth factor receptor [*Mizuhopecten yessoensis*]	-7.5487	0.000887	F > M	Cluster-18098.1;	-1.04369702	0.041341124	F > M	F > M
fem-1	Cluster-4389.1741	sex determining protein Fem-1 like protein [*Pinctada margaritifera*]	7.08	8.23E-06	F > M	Cluster-10581.0;orf1	-0.42988855	0.011276669	-	F < M
foxl2	Cluster-22036.0	forkhead box protein L2-like [*Crassostrea virginica*]	-4.5978	0.000749	-	-			-	F > M
MMP	Cluster-4389.6602	PREDICTED: stromelysin-3 [*Crassostrea gigas*]	-3.0314	0.001984	F > M	Cluster-4389.6602;orf1	-0.59494927	0.421931357	-	F > M
MPI	Cluster-4389.8345	metalloproteinase inhibitor 3 [*Lingula anatina*]	-2.9136	0.000226	F > M	Cluster-20469.0;orf1	-0.61434809	0.310785182	-	F > M
mPRα	Cluster-14850.0	membrane progestin receptor alpha-B-like [*Mizuhopecten yessoensis*]	5.6866	0.000113	F < M	-	-	-	-	F < M
PLAC8	Cluster-4389.12722	PREDICTED: placenta-specific gene 8 protein-like [*Crassostrea gigas*]	4.8244	4.39E-05	F < M	-	-	-	-	F < M
sox2	Cluster-16149.0	Sox2 [*Pinctada fucata*]	-4.7151	2.84E-10	F > M	-	-	-	-	F > M
vitellogenin	Cluster-20609.0	vitellogenin [*Mimachlamys nobilis*]	-9.1347	6.56E-09	F > M	Cluster-20609.0;orf1	-3.14219096	0.14095379	-	F > M

**Fig. 9:**
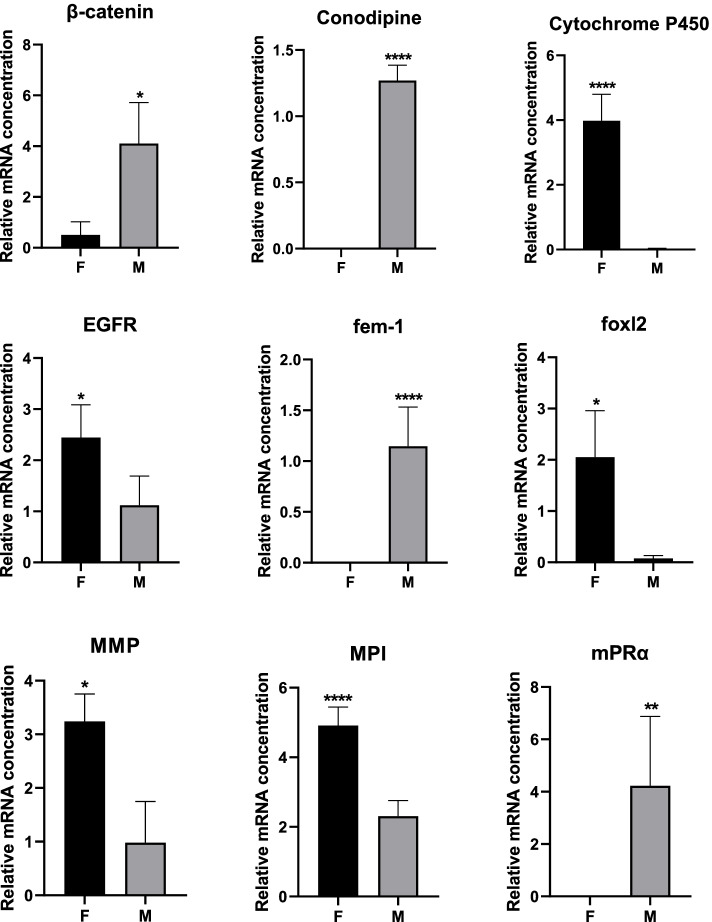
mRNA expression levels for 12 genes in gonads of males and females examined by qPCR to verify the proteome and RNA-Seq data. mRNA levels were quantified by qPCR. 60s RP-L15 and β-actin served as reference genes to normalise expression levels. Results are expressed as fold change (FC). Each bar represents the mean ± standard deviation (SD) of three samples. Significant differences are indicated by an asterisk (*p <0.05, **p <0.01, ***p <0.001, ****p <0.001). EGFR, epidermal growth factor receptor; MMP, matrix metalloproteinase; MPI, metalloproteinase inhibitor; mPRα, membrane progestin receptor alpha; PLAC8, placenta-specific gene 8.

The qPCR results showed that differential expression patterns of 11 of the 12 selected genes were generally consistent with the RNA-Seq analysis (Fig. 9, Table 5); expression of four genes (beta-catenin, conodipin, MPRα and PLAC8) was significantly higher in M compared to F, while expression of eight genes (CYP450, EGFR, fem-1, foxl2, MMP, MPI, sox2 and vitellogenin) was significantly higher in F than M. Therefore, logFC and qPCR assays, RNA-Seq data, and label-free data were correlated. The consistency between qPCR and RNA-Seq confirmed the reliability of RNA-Seq data for accurately quantifying gene expression.

## Discussion

### Determining the Direction of Sex Change based on Histological Characteristics of *G. haimaensis* Gonads

When dissecting gonads of *G. haimaensis*, there was no difference in colour, size or general appearance between males and females; both M and F gonads were white (Fig. 1a). Based on the histological characteristics of gonads, gonads from three females and three males were chosen for transcriptome and proteome analyses.

Spermatogenic cysts were filled with spermatids, some of which were surrounded by follicular cavities and oogonia (Fig. 1a), and 6 of 15 of the examined gonads were testes, all with oogonia. There were no spermatocytes in ovaries, primary oocytes were close to the follicular wall, and they absorbed nutrients from the follicular wall to develop into mature oocytes (Fig. 1b). These characteristics indicate that male *G. haimaensis* may be intersex, and male testis might convert into female gonads.

### Candidate Genes Involved in Sexual Development

Although several sex-related genes have been studied in terms of sex determination and/or differentiation, little information about sex reversal/differentiation cascades is available for *G. haimaensis*.

Some female-biased genes were expressed more highly in M than F, including β-catenin, fem-1, mPRα and PLAC8. β-catenin, which transduces the canonical Wnt signalling pathway in mammals by promoting stability in the cytoplasm and nuclear entry, is critical in female ovary differentiation [41]. In molluscs, β-catenin was expressed mostly in mature female gonads, which indicates a conserved role in the maintenance of ovaries [11, 42]. The ankyrin repeat protein Fem-1 is a component of the signal transduction pathway controlling sex determination [43]. The fem-1 gene of *P. margaritifera* was expressed specifically in different reproductive stages (undetermined, sexual inversion, and regression), suggesting that it may be involved in the sperm-oocyte switch [17]. mPRα was primarily localised on the oocyte plasma membrane, where it regulates induction of oocyte maturation by specifically binding progestins [44]. Progestins exert rapid, multifaceted and nongenomic effects on sperm physiology through mPRα in a variety of vertebrate species [45–47], but few on invertebrates have been reported. In *Octopus vulgaris*, progesterone can also induce activation in spermatozoa via mPRα [48]. However, our qPCR results showed that β-catenin, fem-1 and mPRα were expressed higher in M than F, consistent with the transcriptomic data. The qPCR results of β-catenin, conodipin, fem-1, foxl2, MMP, MPI, mPRα, plac8, sox2 and vitellogenin differed from the proteomic data, which may be because not all transcripts were translated, and mRNA abundance may not correspond to protein expression due to pre-translational, co-translational, or post-translational modification. Combined with the histological characteristics of gonads, we speculate that the males maybe intersex undergoing sex change . A previous study on *Bathymodiolus platifrons* revealed low levels of genetic diversity differences between vent and seep populations [49]. Our results also showed low levels of genetic diversity differences for *G. haimaensis* between vent and seep populations (data not shown, unpublished), which may reflect the survival strategy of sex change that could lead to self-fertilisation to expand the population.

Plac8 is a placental-enriched gene expressed in the spongiotrophoblast layer during mouse development [50]. In mouse, it also plays an important role in spermatocyte differentiation during spermatogenesis [51]. Plac8 has been well studied in vertebrates, yet little is known about its role in invertebrates. In the planarian *Dugesia japonica*, Plac8 plays essential roles in immune responses and development [52]. In *G. haimaensis*, Plac8 was expressed higher in M than F, consistent with the transcriptomic data, implying that Plac8 may be involved in spermatocyte differentiation.

Conodipine-M is a novel phospholipase A2 enzyme isolated from the venom of the marine snail *Conus magus* [53]. Conodipine is secreted by the poison gland in the venom tube and the inner wall of the poison sac. It can specifically act on various ion channels such as potassium, sodium, calcium, and various receptors on the cell membrane, to affect signal transmission in nerve and other cells [53]. Our qPCR results revealed sexual dimorphism for expression of conodipine-M, with significantly higher levels in M than F, suggesting that it may participate in the maintenance of masculinisation. However, the specific role of conodipine-M in testis needs to be clarify in the future.

Some ovary-biased genes reported in other studies and were also identified in our current research. CYP450, EGFR, foxl2, MMP, MPI and vitellogenin were expressed at significantly higher levels in F than M, consistent with the transcriptomic data, and the qPCR results for CYP450 and EGFR were consistent with the proteomic data. In the mammalian female pathway, foxl2 functions by upregulating CYP19A, which encodes the aromatase that converts testosterone into oestrogens [54]. However, CYP19A has been demonstrated to have arisen in the chordate lineage [55], hence it does not exist in bivalves. This finding suggests that despite the possible deep conservation of sex-determining genes among different clades, their regulatory network may have diverged during evolution. The forkhead box L2 gene (foxl2), which encodes a forkhead class transcription factor, is a key factor in the differentiation and maintenance of ovaries in vertebrates. And in molluscs, foxl2 also showed a sexually dimorphic pattern with higher expression levels in females [10, 13, 14, 17, 56], consistent with our qPCR results, which indicates a conserved structure and function of foxl2. Localisation of the Foxl2 protein in spermatogonia and spermatocytes implies that it may also be involved in spermatogenesis in *G. haimaensis.* The EGFR system contributes to key stages of reproduction, such as ovulation, fertilisation, embryo implantation, and the attainment of sexual maturity [57]. Most studies have focused on vertebrates, and few findings have been reported for invertebrates. Our results indicate that EGFR may play a conserved role in ovary development.

Matrix metalloproteinases (MMPs) play an important role in the reproductive process by degrading the extracellular matrix and weakening the follicle wall, leading to follicle rupture [58, 59]. MMP2 and MMP9 stimulate luteinising hormone (LH)-induced steroid production by regulating the release of the EGFR ligand [60]. Tissue inhibitors of metalloproteinases (TIMPs) are proteins secreted by a variety of cells that can selectively inhibit the activity of MMPs. The mechanism of action is to form chelates with metal ions necessary for the active sites of MMPs [61]. Under physiological conditions, TIMPs and MMPs jointly maintain the stability of the biological environment in vivo. However, under pathological conditions, due to their direct action on MMPs, TIMPs are an extremely critical factor for maintaining homeostasis when the activity of MMPs activity is imbalanced. In *G. haimaensis*, MMPs may combine with TIMPs to promote EGFR to stimulate ovary development.

Vitellogenin (VTG), a precursor of yolk protein in oviparous animals, is a molecular carrier that transports nutrients into egg cells. Research on the biological functions of VTG shows that it regulates the osmotic pressure of ovaries [62, 63]. It is also an important immune molecule against pathogenic microorganisms [64–66], and it assists sperm fertilisation [67, 68]. Our transcriptome data and qPCR results showed that vtg was more highly expressed in F than M, indicating an exclusive role in ovaries rather than intersex or testes.

Sox2, a member of the SOX (SRY-related HMG-box) family, is an important transcription factor participating in embryogenesis [69, 70], neurogenesis [71–73], maintenance of stem cells [74–76] and proliferation of primordial germ cells (PGCs) [77]. Also, Sox2 is involved in male germ cell development and stem cell maintenance in fish [78] and spermatogenesis in Zhikong scallop *Chlamys farreri* [79]. In medaka, Sox2 is specifically expressed in ovary [80], and in *Paralichthys olivaceus* Sox2 is expressed more highly in ovary than testis [81]. Our results showed that *sox2* was expressed significantly higher in F than in M, consistent with the transcriptomic data, and localised in both testis and ovary, suggesting that Sox2 may function in male germ cell development and the maintenance of feminisation.

### Putative Mechanism of Sex Change in *G. haimaensis*

In sex determination, the foxl2-leading pathway and RSPO-1/WNT4/β-catenin signalling pathway act independently and complementary to each other to promote and maintain ovarian development [82–85]. In *G. haimaensis*, foxl2 was specifically expressed in ovary, while β-catenin was expressed more highly in testis, implying that the foxl2-leading pathway may perform a leading role in the ovary determination/maintenance pathway of *G. haimaensis.* Also, Foxl2 may upregulate CYP450 to increase estrogens, and MMPs may combine with TIMPs to regulate EGFR and stimulate ovary development. The histological characteristics male gonads resembled intersex gonads, and β-catenin may play a role in intersex gonads to initiate ovary development.

Fem-1, mPRα and PLAC8 are downstream genes of the β-catenin signalling pathway regulated by β-catenin that may be involved in oogenesis in testis. We did not identify a testis-determining factor, such as SRY (Sex Determining Region Y) or DMRT, but there may be other male-determining genes. Sedentary lifestyles, combined with patchy distribution and environmental heterogeneity in darkness and in the presence of high concentrations of heavy metals and other toxic substances may stimulate *G. haimaensis* to change sex and thereby increase reproductive output.

In conclusion, our sex-biased proteomics and transcriptomics analysis of testes and ovaries in *G. haimaensis* revealed a strong correlation between mRNA and protein levels of key genes and proteins. Twelve DEGs between sexes were identified, and four ovary-biased genes (β-catenin, fem-1, foxl2 and mPRα) were expressed significantly higher in M than F. Combining histological characteristics of gonads we speculate that the males maybe intersex undergoing sex change , and male testis might convert into female gonads in *G. haimaensis*. This adaptation may be based on local environmental factors, sedentary lifestyles, and patchy distribution. These findings suggest a deeply conserved function of these genes in sex development, and a diverged regulatory pathway during evolution. This study provides a valuable genetic resource to better understand the mechanisms of sex change and the survival strategies in deep-sea bivalves.

## Supplementary Information

**Additional file 1.**

**Additional file 2.**

**Additional file 3.**

**Additional file 4.**

**Additional file 5.**

**Additional file 6.**

## Data Availability

Data for this manuscript are available at the Science Data Bank (https://www.scidb.cn/s/2MbaA3;https://www.scidb.cn/s/i6N7zy).
