# Supplementary material for: Proteome and Transcriptome Analysis of Gonads Reveals Intersex in Gigantidas haimaensis
Source: BMC Genomics. 2022 Mar 3;23:174. doi: 10.1186/s12864-022-08407-w (PMC8892766; doi:10.1186/s12864-022-08407-w)
Supplement: Supplementary file 1 — Additional file 1. [file 12864_2022_8407_MOESM1_ESM.docx]

Table S1 Transcripts annotated in database

| **Database** | **Number.of.Unigenes** | **Percentage** |
| --- | --- | --- |
| Annotated in NR | 17090 | 40.46 |
| Annotated in NT | 8163 | 19.32 |
| Annotated in KO | 8183 | 19.37 |
| Annotated in SwissProt | 12062 | 28.55 |
| Annotated in PFAM | 14298 | 33.85 |
| Annotated in GO | 14297 | 33.84 |
| Annotated in KOG | 6639 | 15.71 |
| Annotated in all Databases | 1993 | 4.71 |
| Annotated in at least one Database | 23991 | 56.79 |
| Total Unigenes | 42238 | 100 |
